# Extracranial Arteriovenous Malformations Rupture in Pregnancy

**DOI:** 10.7759/cureus.22798

**Published:** 2022-03-03

**Authors:** Khairul Anuar Azis, Khai Luen Koh, Wan Azman Wan Sulaiman, Muath Mamdouh Mahmod Al-Chalabi

**Affiliations:** 1 Reconstructive Sciences Unit, Hospital Universiti Sains Malaysia, Kota Bharu, MYS; 2 Plastic and Reconstructive Surgery, Hospital Kuala Lumpur, Kuala Lumpur, MYS

**Keywords:** ruptured arteriovenous malformations, vascular anomalies, intracranial arteriovenous malformations, extracranial arteriovenous malformations, arteriovenous malformations

## Abstract

Arteriovenous malformations are congenital vascular malformations with a high flow rate. They are made up of a complex vessel system that forms a nidus by connecting feeding arteries to draining veins. Arteriovenous malformations can be fatal due to progressive symptoms and infiltrative disease. The head and neck are the most affected areas by extracranial arteriovenous malformations, followed by the limbs. Hormonal changes during pregnancy lead to the expansion of arteriovenous malformations, which may lead to aggressive progression. We present a case of a patient who had a very rare presentation of ruptured forehead arteriovenous malformations during her fifth pregnancy. A combination of radiological embolization followed by surgical excision was used to treat the condition.

## Introduction

Vascular anomalies are disorders of the endothelium and surrounding cells that can affect the vasculature and involve any anatomical structure. The most common problem associated with vascular anomalies is psychological distress related to the disfigurement as well as functional defects because many lesions affect the head and neck [1]. Local complications (bleeding, infection, obstruction, pain, thrombosis, ulceration, and destroyed anatomic structures) and general complications (congestive heart failure, disseminated intravascular coagulation, pulmonary embolism, thrombocytopenia, and sepsis) can all be caused by vascular anomalies. Vascular malformations are errors in the morphogenetic processes that regulate vascular development between the fourth and 10^th^ weeks of embryonic life. In plastic surgery and interventional radiology, aortic and venous abnormalities of the face are among the most difficult to treat. The treatment of these recurrent lesions necessitates a multidisciplinary approach. After a successful surgical resection and selective embolization, the reconstructive surgeon faces a Herculean task: the restoration of a facial contour that has been severely deformed due to the disruption of several clearly defined aesthetic units [2]. This article reports a 35-year-old Malay lady with a known case of arteriovenous malformations (AVMs) over her forehead who presented with a rupture of the AVMs during the 26^th^ week of gestation, which was surgically excised.

## Case presentation

A 35-year-old Malay woman with a history of AVMs since childhood presented to the emergency department with a history of forehead bleeding. The AVMs had been present since childhood and had been growing since the age of 21. During her previous two pregnancies, the lesion expanded in size and was associated with skin abnormalities, but it shrank postpartum. The lesion was rapidly expanding with obvious skin changes over AVMs. On the 26th week of gestation, the malformations suddenly ruptured without a history of preceding trauma and profusely bled. Manual compression was done to halt the bleeding. However, it was not effective.

**Figure 1:**
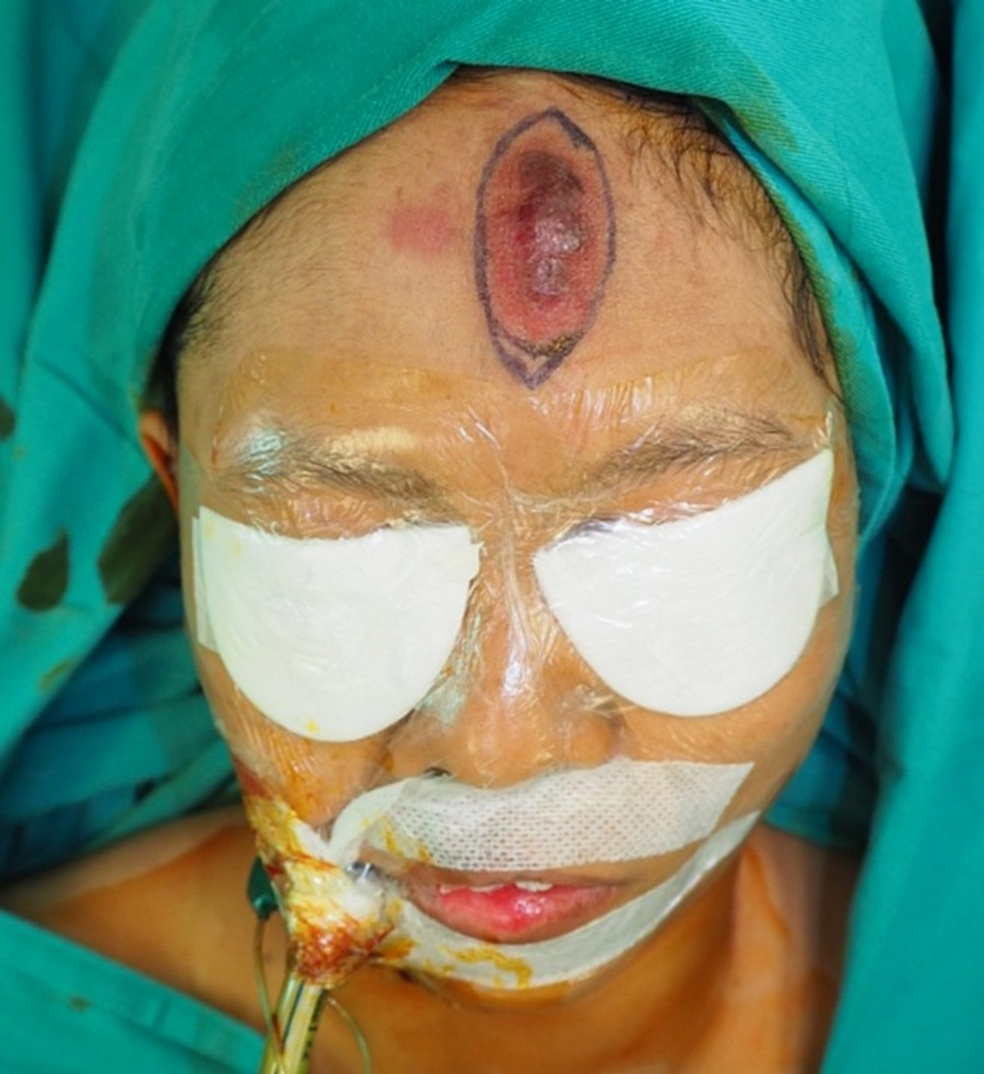
Mid-forehead ruptured extracranial arteriovenous malformation.

Physical examination revealed a hyper-pigmented, raised lesion around 5x2cm, smooth surface, and regular margin on the forehead as shown in Figure 1. The lesion was warm, firm, non-tender, and pulsating. Blood was oozing from the superior part of the lesion.

**Figure 2:**
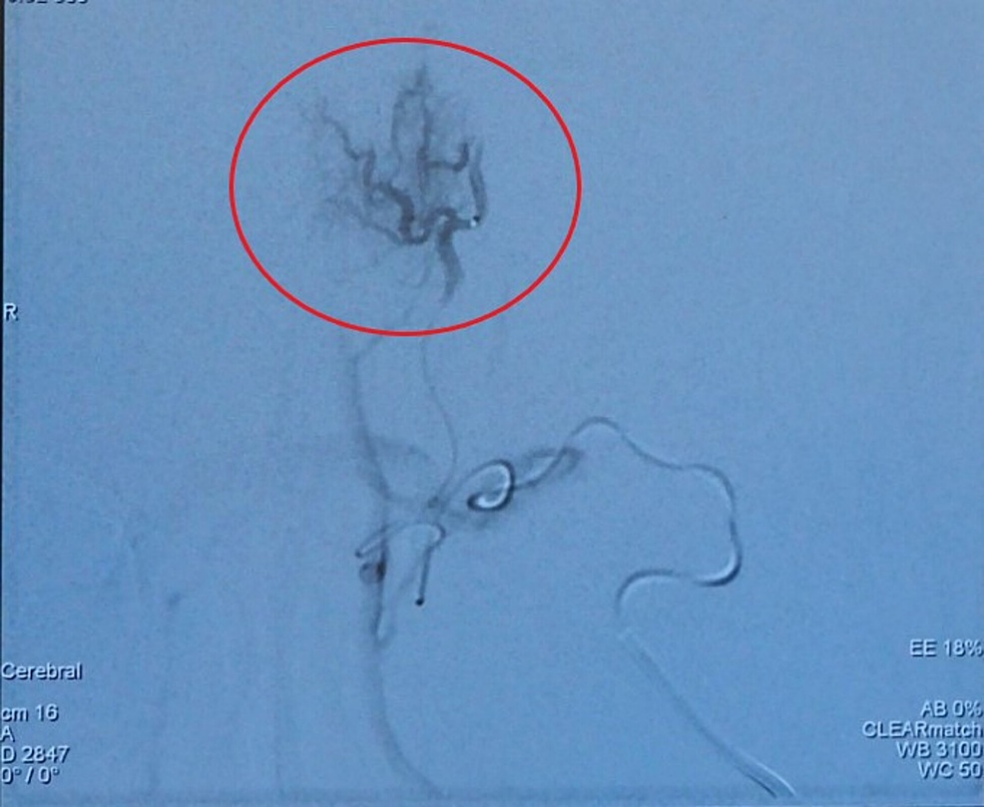
Pre-embolization digital subtracted angiogram showing AVMs are mostly supplied by the terminal branches of the left maxillary artery (red circle).

**Figure 3:**
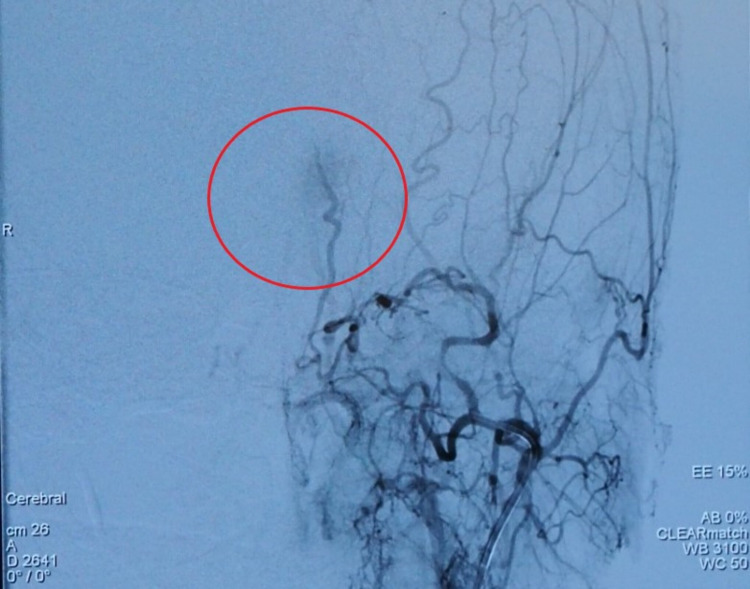
Post-embolization digital subtracted angiogram showing greatly reduced contrast opacification within the arteriovenous malformations (red circle).

Following a multidisciplinary team meeting, we determined that angioembolization before the excision biopsy would have been beneficial to the patient. Angio-embolization was performed, which revealed that the forehead AVMs are mostly supplied by the terminal branches of the left maxillary artery as shown in Figure 2 and that after embolization, contrast opacification within the arteriovenous malformations was greatly reduced, as shown in Figure 3.

**Figure 4:**
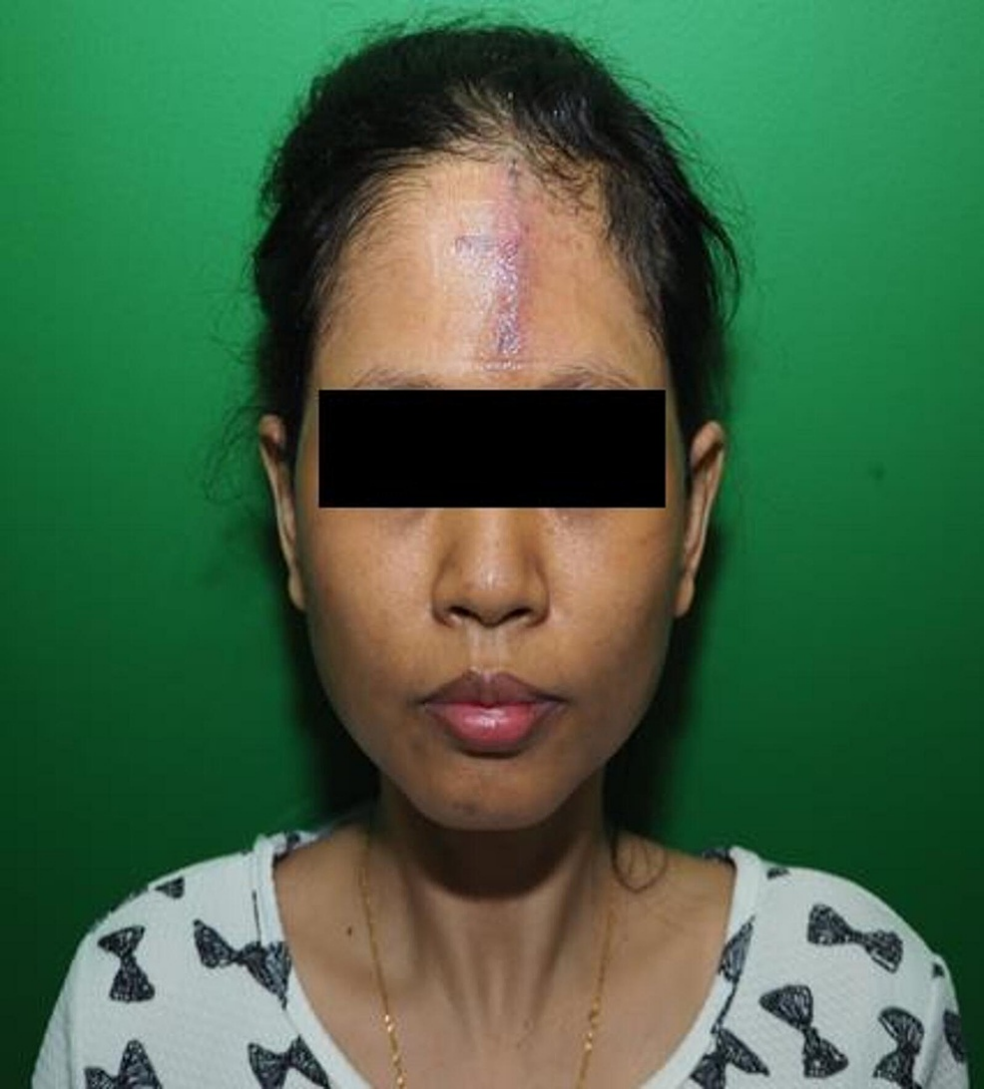
Two weeks post excision.

Surgical excision was performed, and the wound was managed to be close primarily without tension. No complications were observed postoperatively, and the patient was discharged a few days later. On clinic follow-up, the scar appears flat and pink, and there are no signs of residual, as shown in Figure 4.

## Discussion

Feeding arteries, fistulae, or nidus, and draining veins make up AVMs. The nature of connectivity between arteries and veins has an impact on the embolic approach used and the treatment success. Over time, AVMs usually grow more symptomatic [3]. The evolution of AVMs can be documented by the Schobinger Staging System. Typically, many of these lesions grow proportionately throughout childhood and may go unnoticed until rapid expansion occurs as a result of local trauma, attempted resection or ligation, or hormonal changes associated with puberty or pregnancy. The “steal” phenomenon, caused by arteriovenous shunting, diminishes nutritive flow to the skin so that ischemic necrosis may ensue with repeated hemorrhage [4].

AVMs are twenty-fold more common in the intracerebral vasculature than in extra-cerebral sites. The head and neck are the most prevalent sites for extracranial AVMs, followed by the limbs, trunk, and viscera [5]. AVMs in the extracranial space are equally distributed between men and women. AVMs are found in more than half of patients (60%) at birth; interestingly, neonatal presentation occurs more frequently in boys than in girls [6]. Typically, the AVM's ominous nature is undervalued or ignored outright. The AVM that expands and presents as a localized tumor or ulceration throughout childhood is uncommon. Local trauma attempted excision/ligation or hormonal changes linked with puberty or pregnancy can all cause expansion. Some AVMs appear to progress quickly throughout puberty [4]. Malformation affected by pregnancy behaved aggressively. In some patients, there was a partial resolution of the malformation after delivery; however, the lesions remained larger and more symptomatic than in the pre-gravid state. Pregnancy, therefore, represents a definite risk in women with a late onset of arteriovenous malformation [6]. Due to multiple pregnancies whereby hormonal changes influence the nature of the AVMs, the lesion appeared to progressively enlarge and causing disturbing symptoms to the patient.

The treatment of arteriovenous malformations can be risky, and the results can be poor. Selective embolization, surgical excision, or mixes of both are used as therapeutic strategies. Preoperative radiographic preparation is important as it is used to confirm the clinical impression, evaluate the extent of vascular malformation before treatment, identify associated malformations, and differentiate atypical masses. The imaging features of a lesion vary based on its clinical stage and location. Most AVMs are identified by the presence of dilated feeding and draining veins within normal or hypertrophied tissue by utilizing ultrasound, CT, and MRI [7]. The reported cure rates of embolization alone for the definitive treatment of AVMs are (5% to 28%), which are lower than the cure rates of surgical resection (80% to 95%) and radiosurgery (65% to 85%) [2]. When employed before surgical resection, however, the value of endovascular embolization becomes clearer. In such circumstances, embolization usually minimizes the size of the AVM and may increase the safety of the resection.

Embolism aims to close the smallest of vessels from the inside out by injecting embolic material into the malformation's epicenter. However, selective embolization with occlusion of one feeding vessel causes immediately increased flow from other feeding vessels. To permanently collapse the major arteriovenous malformations, all micro-fistulas and macro-fistulas must be eliminated. Preoperative embolization is indicated to reduce blood flow and facilitate surgical excision but should not be used to reduce the extent of resection [8]. The only way to be cured is to have the tumor completely removed. Beyond the margins of resection, leaving dormant or residual arteriovenous channels stimulates additional collateralization, shunting, and re-expansion. Resection of AVMs should be done 24 to 48 hours post embolization or there is a risk of revascularization from feeding vessels. For severe high-flow vascular abnormalities, simultaneous preoperative embolization and resection can produce excellent results.

The goal of surgery is total removal of the nidus of the AVMs as leaving behind residual or dormant anomalous channels may lead to further collateral formation, shunting, and expansion. As the overlying skin was involved, it was excised. Before resection, options for closure were considered. As the lesion was small and well-localized, a primary closure could be achieved. Another form of primary closure is either a skin graft or a local or microvascular flap. Delayed primary closure with a skin graft is particularly useful if bleeding is excessive, provided that vital structures are not exposed. Abnormal collateral vessels may be present after primary or delayed covering with a local cutaneous or musculocutaneous flap. If the AVMs are not completely removed, the abnormality will recur and may entangle the skin graft or flap. Hurwitz and Kerber in 1981 proposed that a microvascular free flap would improve the local hemodynamic state at the site of incompletely resected AVMs and thus minimize the likelihood of dilatation, collateral formation, and reappearance [4]. Post-operatively the patient was monitored for any complications of post embolization such as tissue necrosis, hematoma, and pulmonary embolism. Follow-up was given to monitor the possibility of recurrence.

## Conclusions

Pregnancy tends to accelerate the nature of AVMs' progression, which can lead to undesirable complications, especially in the head and neck region. AVMs that rupture during pregnancy can be treated similarly to those that occur in non-gravid women. Embolization followed by surgical excision offers a higher cure rate and reduces complications intra-operatively.
